# Human Granuloma *In Vitro* Model, for TB Dormancy and Resuscitation

**DOI:** 10.1371/journal.pone.0053657

**Published:** 2013-01-07

**Authors:** Nidhi Kapoor, Santosh Pawar, Tatiana D. Sirakova, Chirajyoti Deb, William L. Warren, Pappachan E. Kolattukudy

**Affiliations:** 1 Burnett School of Biomedical Sciences, College of Medicine, University of Central Florida, Orlando, Florida, United States of America; 2 Sanofi Pasteur, VaxDesign Campus, Orlando, Florida, United States of America; Fundació Institut d’Investigació en Ciències de la Salut Germans Trias i Pujol. Universitat Autònoma de Barcelona. CIBERES, Spain

## Abstract

Tuberculosis (TB) is responsible for death of nearly two million people in the world annually. Upon infection, *Mycobacterium tuberculosis* (*Mtb)* causes formation of granuloma where the pathogen goes into dormant state and can live for decades before resuscitation to develop active disease when the immune system of the host is weakened and/or suppressed. In an attempt to better understand host-pathogen interactions, several groups have been developing *in vitro* models of human tuberculosis granuloma. However, to date, an *in vitro* granuloma model in which *Mtb* goes into dormancy and can subsequently resuscitate under conditions that mimic weakening of the immune system has not been reported. We describe the development of a biomimetic *in vitro* model of human tuberculosis granuloma using human primary leukocytes, in which the *Mtb* exhibited characteristics of dormant mycobacteria as demonstrated by (1) loss of acid-fastness, (2) accumulation of lipid bodies (3) development of rifampicin-tolerance and (4) gene expression changes. Further, when these micro granulomas were treated with immunosuppressant anti-tumor necrosis factor-alpha monoclonal antibodies (anti-TNFα mAbs), resuscitation of *Mtb* was observed as has been found in humans. In this human *in vitro* granuloma model triacylglycerol synthase 1deletion mutant (Δ*tgs1*) with impaired ability to accumulate triacylglycerides (TG), but not the complemented mutant, could not go into dormancy. Deletion mutant of *lipY*, with compromised ability to mobilize the stored TG, but not the complemented mutant, was unable to come out of dormancy upon treatment with anti-TNFα mAbs. In conclusion, we have developed an *in vitro* human tuberculosis granuloma model that largely exhibits functional features of dormancy and resuscitation observed in human tuberculosis.

## Introduction

Tuberculosis (TB) caused by *Mycobacterium tuberculosis (Mtb)*, remains a major threat to the world population as one-third of the world population is latently infected. Upon infection, only 5% of people develop active TB, whereas majority of people carry a lifelong latent infection. *Mtb* enters the host *via* aerosolization, where it infects and activates macrophages and dendritic cells in the lungs. The activated dendritic cells, present the processed antigens to CD4 T cells [1]. These activated lymphocytes and infected macrophages, in response to inflammatory cytokines and chemokines, migrate to the site of infection where they can form organized structures called granulomas in which *Mtb* goes into a drug-resistant dormant state. In latent TB infection, humans can harbor a small number of dormant *Mtb* bacilli that are likely contained in microgranuloma. These organisms are viable but in a dormant state.

Animal models have been used to study various aspects of granuloma formation, dormancy and the host-pathogen interactions. Mice is not the natural host of *Mtb;* granuloma in mice have a different cellular organization than in humans [2], [3]. Rabbit and guinea-pig models also do not exhibit the full spectrum of the human TB disease [4]. Even though non-human primate models more closely resemble the various manifestations of human TB [5] they are prohibitively expensive to maintain under BSL3 laboratory conditions.

Several groups have attempted to develop *in vitro* models of granuloma [6]–[7]. For example, an *in vitro* granuloma model was developed to study the molecular interactions between mycobacteria and human host cells using mycobacterial antigen coated sepharose beads, or live mycobacteria, to induce granuloma formation with human peripheral blood mononuclear cells (PBMCs) [8]–[12]. The *Mtb* in this model was a good step towards using culture methods to study TB, but did not exhibit features of dormancy. In a lipid-loaded macrophage model *Mtb* has been shown to go into a dormant state [13]. However resuscitation has not been demonstrated in any human cell system. When the host immune system is weakened, it is well known that dormant *Mtb* resuscitates leading to active TB. Experimental evidence has revealed that tumor necrosis factor (TNFα) plays a major role in host defense against *Mtb* in both the active and chronic phases of infection, [14]–[18]. Data suggest that some TNF activity is required to maintain the integrity of the granuloma and to confine the TB pathogen [17], [19]. An *in vitro* human granuloma model in which *Mtb* goes into a drug-resistant dormant state and resuscitates upon conditions that mimic immune suppression provides an opportunity to study both mycobacterial dormancy and potential resuscitation that may occur with immunosuppressant therapies. Our aim was to develop a biomimetic model of latent TB that could be used to accurately reflect both granuloma dormancy and reactivation.

In this report we present an *in vitro* model of human TB granuloma and demonstrate the development of *Mtb* dormancy in the granuloma and resuscitation upon immune suppression caused by anti-TNFα mAb treatment. We demonstrate that deletion of *tgs1* (*Rv3130*), that has been strongly implicated in the development of dormancy, prevented *Mtb* from going into dormancy and deletion of *lipY (Rv3097c)*, thought to be involved in mobilization of stored TG, prevented resuscitation caused by anti-TNFα mAb treatment. Thus, the *in vitro* granuloma model emulates many TB features observed in human patients.

## Results

### Infection of Human PBMCs Resulted in the Formation of 3D Granuloma

**Figure 1:**
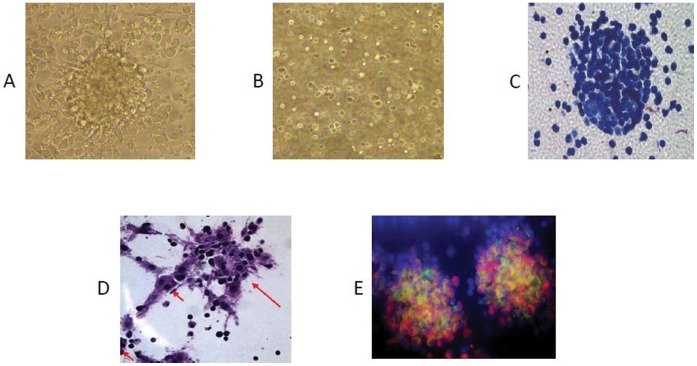
Infection of human PBMC with *Mycobacterium tuberculosis* resulted in the formation of microscopic granulomas. (**A**) Infected PBMCs, (**B**) uninfected PBMCs, (**C**) H & E staining showing micro granulomas, (**D**) H & E staining showing multinucleated giant cells. (**E**) Fluorescent staining of granulomas sections with DAPI (nuclear stain), CD68 (macrophage marker-shown in red) and CD3 (T cells-shown in green) monoclonal antibodies.

To replicate dormant TB in an *in vitro* model, we infected human PBMCs placed in a collagen matrix with *Mtb* H37Rv and incubated for 8 days. PBMCs tended to form microscopic granulomas (micro-granuloma) at multiplicity of infection (MOI) 1∶0.1, as observed from aggregation of lymphocytes surrounding infected macrophages (Fig. 1A). Corresponding control uninfected samples from the same donors did not form these aggregates (Fig. 1B) indicating that aggregation forms in response to *Mtb* infection. At a lower MOI of 1∶0.05, microscopic granulomas could not be observed; for MOI of 1∶1or higher, the *Mtb* cells tended to induce lysis of the host cells (data not shown). The granuloma-like shape of the cell aggregates formed following *Mtb* infection was confirmed by histology. Granuloma samples exhibited aggregation of lymphocytes around the macrophages (Fig. 1C). We also observed formation of multinucleated giant cells (Fig. 1D, arrows show multinucleated cells) which are a recognized characteristic of tuberculosis granuloma. [10]. To identify the cellular components of the *in vitro* grown granuloma immunohistochemical examination was performed using Fluorescent CD68 (macrophage marker) and CD3 (T cells-shown) mAbs. The micro granulomas were positive for both CD68 and CD3 antibodies, thus providing further evidence that the granulomas consisted of both T cells and macrophages (Fig. 1E).

### *Mtb* Infection Resulted in a Decrease in Macrophage and CD4+ T Cell Number

**Figure 2:**
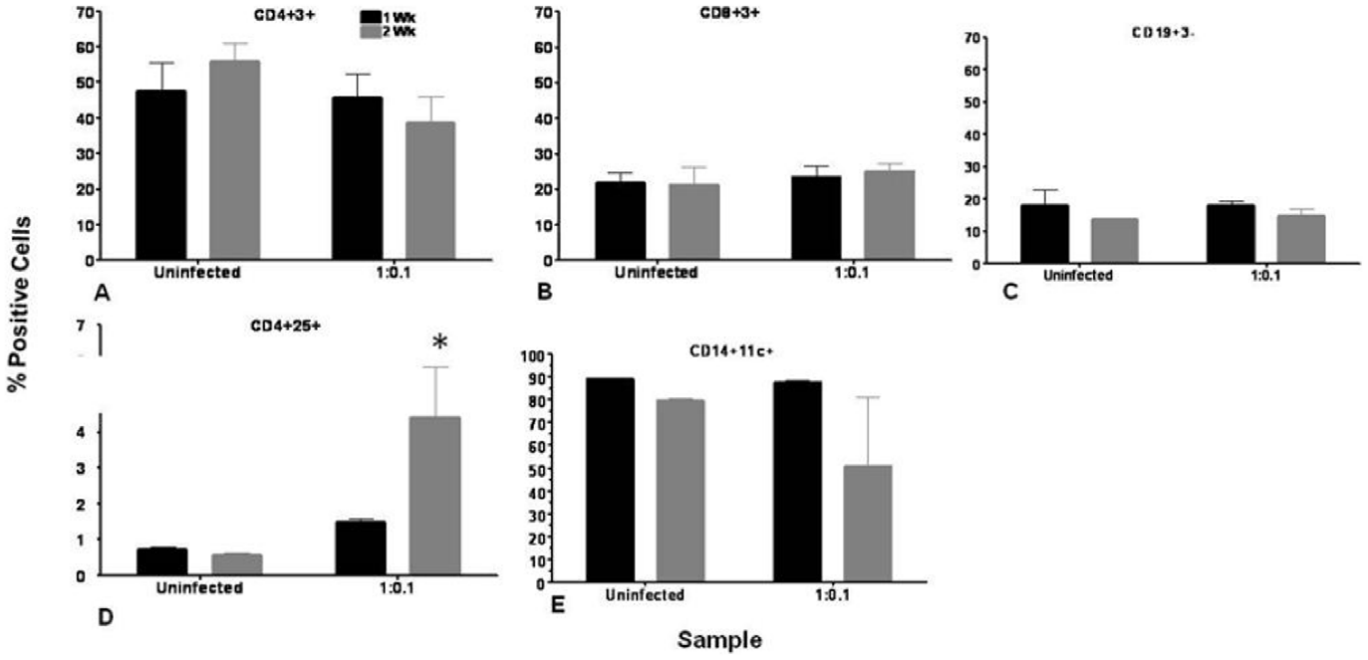
*Mtb* infection resulted in a decrease in macrophage and CD4+ T cell number. Granuloma samples were harvested and host cells were analyzed for expression of cellular markers by flow cytometry as described in methods. Graphs depict 1 wk and 2 wk post-infection profiles of uninfected and infected granuloma samples for (**A**) CD4+3+ T cells, (**B**) CD8+3+ T cells, (**C**) CD19+ B cells, (**D**) CD4+25+ T cells, (**E**) CD14+11c+ macrophages. Data are represented as mean +/− SEM from 3 experiments.* p<0.05 for CD4+25+ cells at 2 wk time point for 1∶0.1 MOI-infected group compared to the uninfected group.

It has been reported that TB causes CD4 lymphocytopenia and is associated with normal numbers of CD8 T cells [20]. In order to test whether our model manifests a similar response, we determined the changes in expression of cellular markers on host cells in response to low-dose *Mtb* infection by flow cytometric analysis. At1 wk and 2 wk post-infection, host PBMC cells were isolated, from infected and uninfected cultures and stained with antibodies as described in methods. Flow cytometric analysis was performed for CD4+ and CD8+ T cells, B cells and monocytic populations as shown in Figure 2. While there was an increase in CD4+3+ T cells in uninfected samples at 2 wks (55.9%±5.2%) compared to 1 wk (44.8%±8%), the inverse was found for infected samples. A decline in numbers of these cells was observed at longer culture times (45.6%±6.7% at 1 wk and 38.8%±7.1% at 2 wk) (Fig. 2A). The CD8+3+ T cell as well as CD19+3- B cell population remained relatively unchanged between uninfected and *Mtb*-infected samples and also between 1 wk and 2 wk samples (Fig. 2B, 2C). CD4+25+ T cells, which are ascribed to regulatory T cells, are known to be involved in immunity to *Mtb*. Figure 2D shows the relative changes in CD4+25+ T cells for infected and uninfected cultures. A three-fold increase was observed in the regulatory CD4+25+ T cell population upon *Mtb* infection at 2 wk compared to 1 wk (1.5%±0.1% at 1 wk and 4.4%±1.4% at 2 wk). The change in macrophage cell numbers is shown in Fig. 2E. There was a large reduction in numbers of CD14+CD11c+ macrophages at 2 wk (50.8%±29.8%) compared to 1 wk (87.6%±0.8%) in response to *Mtb* infection. No decline in macrophage numbers was observed in uninfected samples at 2 wk compared to 1 wk.

### Infection of Human PBMCs with *Mtb* Induces Inflammatory Cytokines

**Figure 3:**
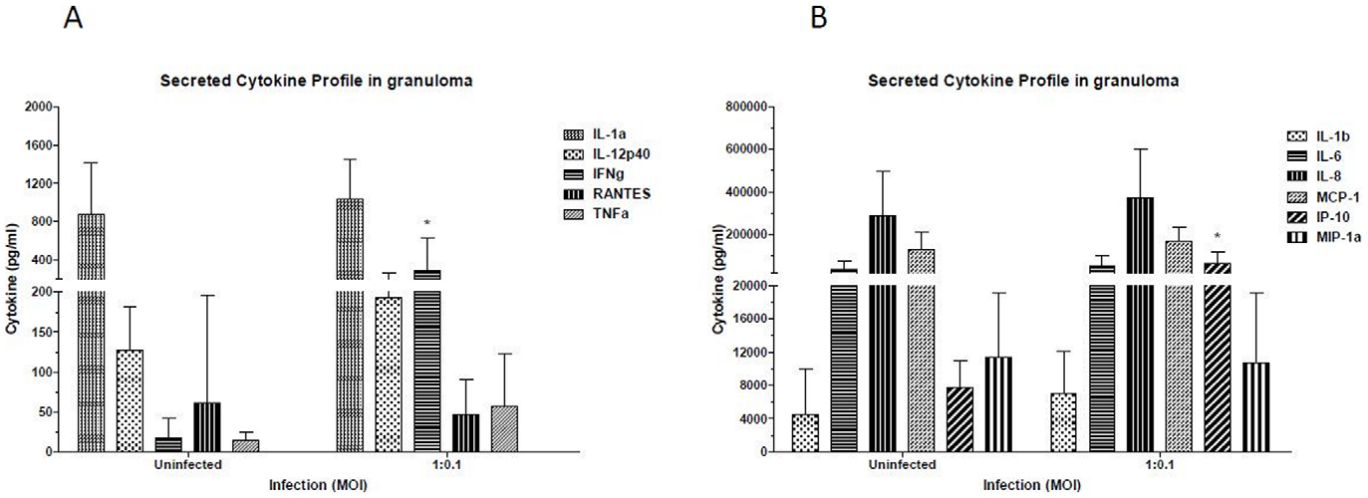
Infection of human PBMCs with *Mtb* induces inflammatory Cytokines. Supernatants from granuloma samples or control uninfected samples were saved at 8-day post-infection and cytokine/chemokine profile determined by multiplex cytokine analysis as described in methods. Profiles for prominent cytokines and chemokine IL-1α, IL-12p40, IFN-γ, RANTES and TNFα are shown in Figure 3A, whereas Cytokines and chemokines IL-1β, IL-8, IL-6, MCP-1, IP-10, MIP-1α are shown in Figure 3B. Data are represented as mean +/− SEM from 3 experiments.

It is known that *Mtb* strongly induces cytokine production during infection [21]. To determine the cytokine/chemokine secretion profile of host cells in response to *Mtb* infection, the culture supernatants from 8–9 day granuloma samples were used for cytokine/chemokine analysis using a multiplex cytokine assay. IFN-γ, TNFα, IL-12p40 (Fig. 3A) and IP-10 (Fig. 3B) were induced by *Mtb* infection. IP-10 is induced via IFN-γ and TNFα, so it is not surprising to observe this chemokine along with IFN-γ and TNFα production.

### *Mtb* within the Granuloma goes into a Dormant State

#### Auramine-O and nile red staining

**Figure 4:**
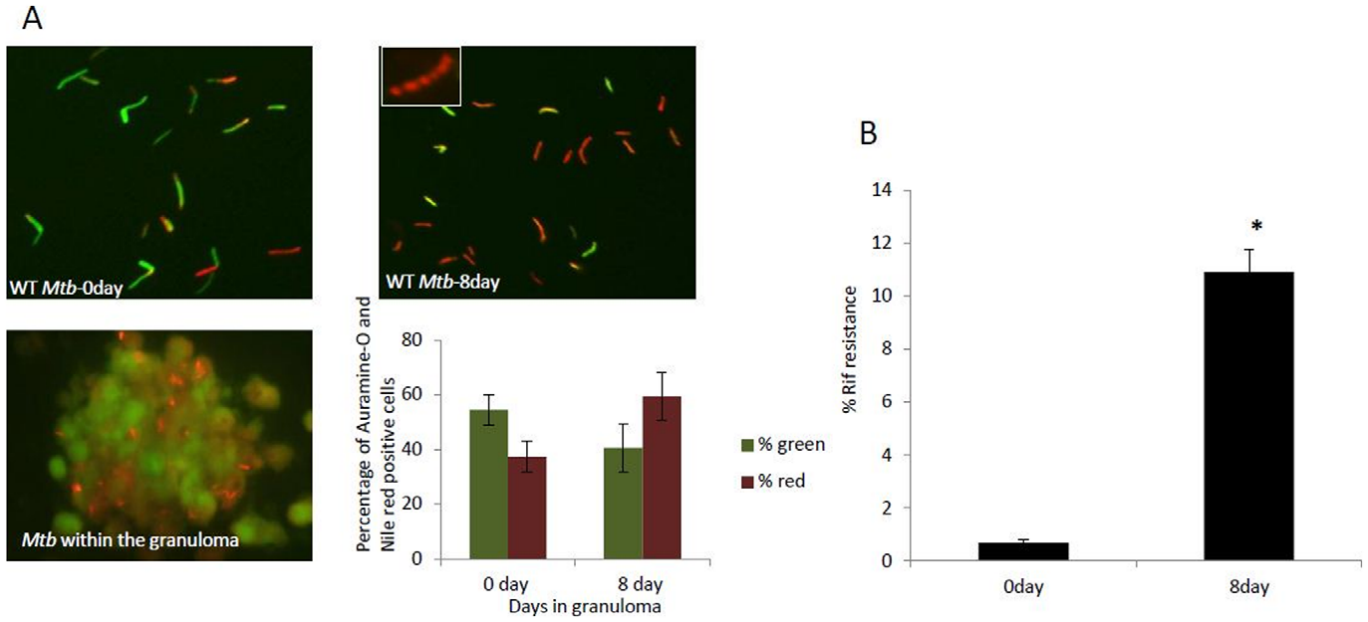
*Mtb* within the granuloma goes into a dormant state. (**A**) Auramine-O and Nile red staining of granuloma sections shows Nile Red positive *Mtb* cells within the granuloma (figure 4A lower left panel, insert in 4A shows lipid bodies). The number of Auramine-O and Nile red positive *Mtb* cells were counted from multiple fields, and percentage of Auramine-O (green) and Nile red (red) positive *Mtb* cells are presented graphically (Figure 4A lower right panel) (**B**) *Mtb* from 8 day granuloma was resistant to Rif when compared to 0 day *in vitro* grown *Mtb*. Data are represented as mean +/− SEM from 3 experiments. P values were calculated using students t test. * p<0.05 for Rif resistance of *Mtb* from 8day granuloma compared to *Mtb* from 0day in vitro grown *Mtb*.

Dormant mycobacteria are known to exhibit loss of acid-fastness and accumulate lipid bodies as they go into dormancy [22]–[27]. To test whether *Mtb* in the *in vitro* granuloma manifests such a dormant phenotype, granuloma sections and *Mtb* cells from 0 day and 8 day granuloma were dual stained with Auramine-O (acid-fast staining of *Mtb* where cells stain fluorescent green) and Nile Red (lipid-body containing *Mtb* cells stain fluorescent red). When granuloma sections were stained, *Mtb* cells could be observed residing within the granulomas (Fig. 4A, lower left panel). After 8 days the host cells were lysed and *Mtb* cells were obtained from the granulomas. It was found that *Mtb* cells had accumulated lipid bodies as indicated by positive Nile red staining (Fig. 4A, top right, lipid bodies can be seen in the inset) when compared to day 0. At day 0 *Mtb* cells exhibit less Nile red but more Auramine-O stained positive cells (Fig. 4A, top left). *In vitro Mtb-*infected granulomas at day 8 had a higher percentage of red stained cells as compared to day 0 *Mtb* samples (Fig. 4A, lower right panel).

#### Rifampicin resistance

Literatures on Mtb dormancy indicates that *Mtb* goes into a dormant state in the granuloma *in vivo* [28]. Rifampicin (Rif)-tolerance is a characteristic of dormant *Mtb* [24], [29]–[31]. Approximately 10% of the *Mtb* from day 8 granuloma samples exhibit phenotypic Rif resistance, as compared to less than 0.5% in the day 0 culture (Fig. 4B). The development of this drug resistance phenotype strengthens the conclusion that our *in vitro* granuloma model accurately reflects TB granulomas in humans.

### Treatment of Granulomas with Anti TNFα Monoclonal Antibody Caused Reactivation of Latent *Mtb*

**Figure 5:**
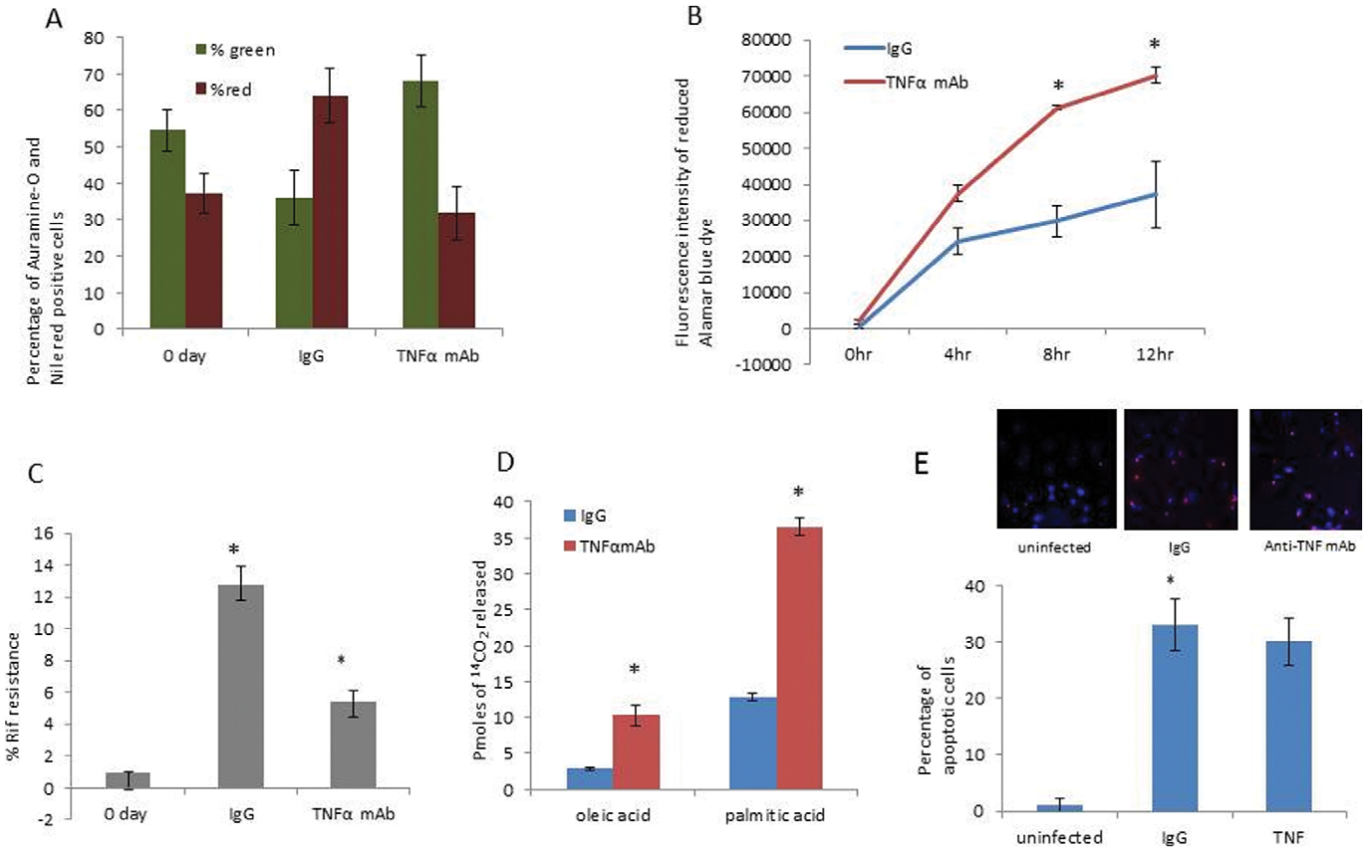
Addition of anti-TNF α monoclonal antibody caused reactivation of dormant *Mtb*. (**A**) Auramine-O and Nile red staining of WT *Mtb* from IgG and anti-TNF α antibody treated granulomas. (**B**) Alamar Blue readouts at different time intervals when WT *Mtb* cells from granulomas treated with anti-TNF-α antibody or control IgG, were incubated with Alamar blue dye. (data of one representative data set has been displayed.) (**C**) WT *Mtb* cells from granulomas treated with anti-TNF-α antibody had a lower antibiotic resistance as compared to WT *Mtb* cells from control IgG treated granulomas. (**D**) *Mtb* cells from granulomas treated with anti-TNF α antibody catabolized fatty acids at a higher rate than *Mtb* from control IgG treated granuloma. P values were calculated using students t test. p<0.05 for metabolism of oleic and palmitic acid for *Mtb* from anti-TNF-αantibody treated granuloma compared to *Mtb* from IgG treated granuloma. (E) Infection of hPBMCs resulted in Apoptosis of these cells. Data are presented as mean +/− SEM from 3 experiments. P values were calculated using students t test. * p<0.05.

TNFα is known to be involved in the formation and maintenance of the granuloma [17], [19]. Neutralizing TNFα or its activity tends to disrupt the granuloma structure *in vivo* allowing *Mtb* to emerge from dormancy and develop active TB [14]–[16], [18], [32], [33]. We tested whether the *Mtb* cells in the *in vitro* granuloma can be resuscitated by anti-TNFαmonoclonal antibody treatment. Once the microgranuloma structures were formed, anti-TNF-α mAbs were added to the media to assess if resuscitation could be observed in vitro. Resuscitation was monitored via changes in dormancy phenotypes, namely- Auramine-O and Nile red staining pattern, and Rif resistance after 6 days of anti-TNFα mAb treatment. Granulomas treated with a control IgG were used for comparison. *Mtb* cells from granulomas treated with the control IgG showed that majority of the cells remained positive for Nile red, and fewer were positive for the Auramine-O stain (Fig. 5A) as expected. However, a great majority of the *Mtb* cells from granulomas treated with anti-TNFα mAbs were positive for the Auramine-O, (Fig. 5A). *Mtb* from granulomas treated with anti-TNFα mAbs also exhibit significantly less Rif tolerance than *Mtb* from granuloma treated with the control IgG. (Fig. 5C).

#### Metabolic activity of *Mtb*

Alamar blue is a cell viability indicator that uses the natural reducing power of living cells to convert resazurin to bright red fluorescent molecule, resorufin, thereby generating a quantitative measure of metabolic activity. *Mtb* obtained from granulomas treated with the anti-TNFα mAbs were more metabolically active as compared to *Mtb* from granulomas treated with the control IgG (Fig. 5B) consistent with reactivation of the granuloma.

Since fatty acids are thought to be the main source of energy, we tested for the rate of catabolism of oleic acid and palmitic acid by *Mtb* from granulomas treated with the control IgG or anti-TNFα mAbs. As indicated by the amount of ^14^CO_2_ released from [1-^14^C] labeled oleic and palmitic acid, we found that the *Mtb* from granulomas treated with anti-TNFα mAbs catabolized oleic and palmitic acid at a higher rate (Fig. 5D) as compared to the *Mtb* from granulomas treated with the control IgG.

### Apoptosis Assay

It has been shown that mycobacterium tuberculosis infection leads to apoptosis of macrophages and is mediated by TNFα [34]. In order to determine if *Mtb* caused apoptosis, PBMCs were infected with *Mtb* H37Rv and apoptosis was determined in uninfected PBMCs and after treatment of granulomas with IgG and anti-TNFα mAb. DNA cleavage in apoptotic cells were detected *in situ* in fixed cells utilizing terminal deoxynucleotidyl transferase (TdT) by TUNEL assay. As assayed by the TUNEL method, infection of *Mtb* resulted in apoptosis of human PBMCs, however treatment of granulomas with anti-TNFα mAb, did not result in any change in the number of apoptotic cells when compared with granulomas treated with control IgG (figure 5E).

### Effect of Anti-TNFα mAb Treatment on Changes in Gene Expression Relevant to Dormancy and Resuscitation

**Figure 6:**
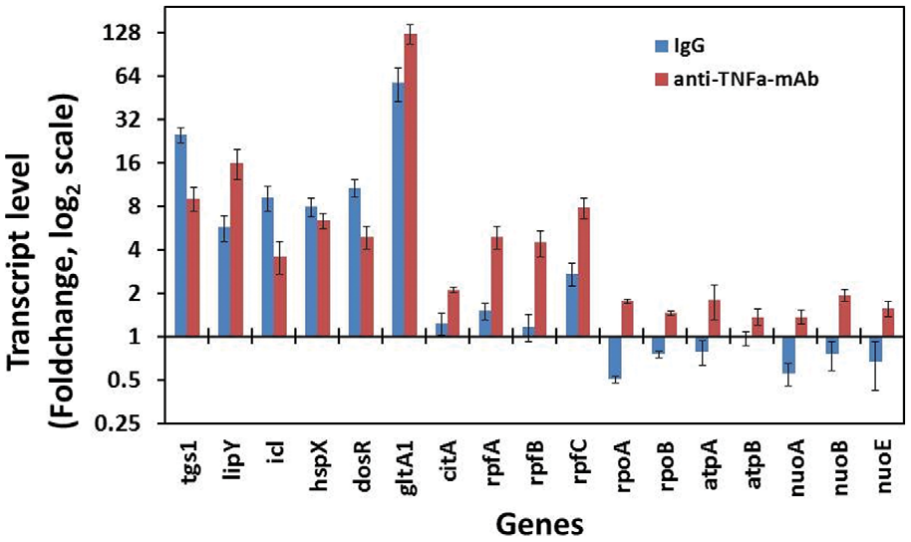
Gene expression profile of *Mtb* cells from *in vitro* granuloma treated with IgG anti-TNFα-mAb. Relative gene expression values (fold changes of day 0) in IgG and anti-TNFα mAb treated Mtb cells are represented for a selected set of genes that are known to be involved in dormancy and resuscitation conditions. Real-time Taqman RT-PCR measurement was performed to measure relative abundance of transcripts. Relative quantitation method (ddCt) was used to determine the fold change in transcripts level. Gene transcript level is expressed as fold change in log2 scale relative to the sample from in vitro grown starter culture used for infection of PBMC. Samples of starter culture (day 0) was used as calibrator and 16S rRNA gene was used as the endogenous control to normalize the expression values. tgs1, triacylglycerol synthase1; lipY, lipase Y; icl, isocitrate lyase; hspX, heat-shock protein X; dosR, dormancy response regulator; gltA1, citrate synthase 1; citA, citrate synthase II; rpf, resuscitation promoting factor (A, B, and C); rpo, RNA polymerase (A and B); atp, ATP synthase (A and B subunits); nuo, NADH dehydrogenase (A, B and E subunits).

*Mtb* cells recovered from *in vitro* granuloma showed dormancy related phenotypes. When the *Mtb* cells within the *in vitro* granuloma were treated with anti-TNFα mAb it demonstrated resuscitation of *Mtb* cells from the *in vitro* dormant state [IgG treated]. To determine whether the observed dormancy and resuscitation related phenotypes are reflected in gene expression changes, we examined the transcript levels of a few selected genes that are mainly involved in storage lipid synthesis, lipid utilization, catabolism, energy generation and transcription based on our previous studies and other reports [13], [35], [36]. Transcript levels of the *tgs1*, *lipY*, *icl* (isocitrate lyase), *dosR*, *hspX* and *gltA1* (citrate synthase I of TCA cycle; Rv1131) were significantly up regulated in the dormant *Mtb* cells (IgG treated cells). *lipY* transcript level was up regulated after anti-TNFα mAb treatment as might be expected from its postulated role in lipid utilization required for resuscitation. The *citA* (citrate synthase II; Rv0889c) and rpfA, rpfB and rpfC [resuscitation-promoting factor genes A (Rv0867c), B (Rv1009) and C (Rv1884c)] were up regulated at a lower level. In the IgG treated cells *rpoA* and *rpoB* (RNA polymerase genes; Rv3457c and Rv0667, respectively); *atpA* and *atpB* (ATP synthase genes; Rv1308, Rv1304 respectively); *nuoA, nuoB* and *nuoE* (NADH dehydrogenase I subunit genes; Rv3145, Rv3146 and Rv3149 respectively) were found to be down-regulated under dormancy inducing condition (IgG treated) but up regulated under resuscitation inducing condition (anti-TNFα mAb treated) Fig. 6.

### *tgs*1 Deletion Compromised the Ability of *Mtb* to go into a Dormant State in the *in vitro* Human Granuloma Model

If *Mtb* utilizes fatty acids as a major source of energy as reported [13], [37], and if *tgs*1, that is known to be the primary contributor to TAG synthesis, is involved, then deletion of tgs1 (Δ*tgs*1), should compromise the ability of the mutant to go into dormancy in the *in vitro* granuloma model. To test for this possibility PBMCs from human donors were infected with *Mtb* WT Δ*tgs*1 mutant and Δ*tgs*1complemented strain (Δ*tgs*1 C+) in a collagen matrix. WT and Δ*tgs*1 mutant and Δ*tgs*1 C+ formed micro-granulomas. The granulomas were then treated with either the control IgG or anti-TNFα mAbs for 6 days, and *Mtb* cells were obtained from the granulomas and assessed for dormancy.

**Figure 7:**
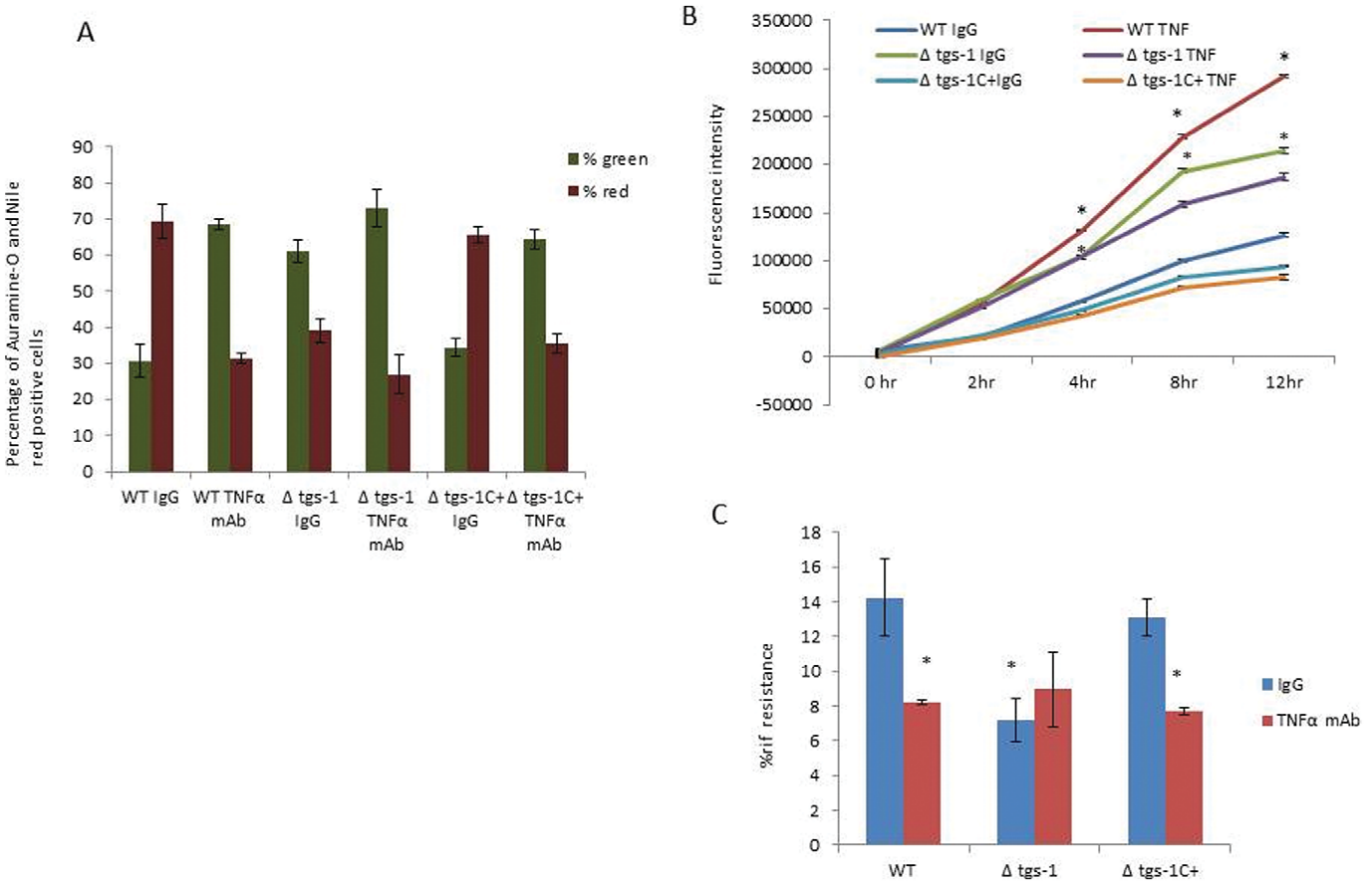
*tgs1* deletion compromised the ability of the *Mtb* to go into a dormant state. (**A**) Auramine-O and Nile red staining of WT and *Δ-tgs1 mutant and Δ-tgs1 complemented strain (Δ-tgs1C+)* population in *in vitro* granuloma. (**B**) Alamar Blue readouts at different time intervals when WT and *Δ-tgs1* mutant *and Δ-tgs1 complemented strain* from granulomas treated with anti-TNF-α antibody or IgG, were incubated with Alamar blue dye. (data of one representative data set has been displayed.) (**C**) *Δ-tgs1* mutant from granulomas treated with IgG or anti-TNF-α antibody had a lower antibiotic resistance as compared to WT *Mtb* cells from IgG treated granulomas. Data are presented as mean +/− SEM from 3 experiments. P values were calculated using students t test. *p<0.05.

Auramine-O and Nile red staining of WT *Mtb* cells from granulomas treated with the control IgG, showed that most of the cells remained positive for Nile red and that most of the WT *Mtb* cells from granulomas treated with anti-TNFα mAb were positive for the Auramine-O stain (Fig 7A). Were as staining of Δ*tgs*1 *Mtb* cells from granulomas treated with IgG, as well as those from granulomas treated with anti-TNFα mAb showed that most of the cells were positive for Auramine-O (Fig. 7A), indicating that the Δ*tgs*1 mutant *Mtb* cells accumulated less lipids as compared to WT *Mtb*. Staining of Δ*tgs*1 C+ *Mtb* cells showed that most of the Δ*tgs*1 C+ *Mtb* cells from granulomas treated with anti-TNFα mAb were positive for the Auramine-O stain indicating resuscitation, whereas most of the cells from granulomas treated with the control IgG, remained positive for Nile red indicating dormancy.

Alamar blue assay results suggest that WT *Mtb* cells obtained from granulomas treated with anti-TNFα mAb and Δ*tgs*1 mutant *Mtb* cells from granulomas treated with IgG or anti-TNFα antibody were more metabolically active as compared to WT *Mtb* from granulomas treated with control IgG (Fig 7B).

WT *Mtb* from granulomas treated with IgG had a higher percentage of Rif resistance as compared to the WT *Mtb* from granulomas treated with anti-TNFα mAbs as well as Δ*tgs*1 mutant *Mtb* cells from granulomas treated with either IgG or anti-TNFα mAb (Fig 7C). Δ*tgs*1 C+ *Mtb* from granulomas treated with anti-TNFα mAbs had a lower Rif resistance as compared to Δ*tgs*1 C+ *Mtb* from granulomas treated with control IgG. All of these results strongly suggest that *tgs*1 deletion compromised the ability of the pathogen to go into dormancy.

### Deletion of *lipY* Compromised the Ability of *Mtb* within the *in vitro* Granuloma to Come out of Dormancy upon Treatment with Anti TNF-α mAb

When the bacterium resuscitates, it utilizes the stored TG as its energy source [38]. It is thought that *lipY* is involved in hydrolyzing stored TAG as the pathogen emerges from dormancy. It has been reported that *lipY*-disrupted mutant (Δ-*lipY*) loses its ability to utilize stored TG. [36]. If *Mtb* utilizes *lipY* to mobilize stored TG to break dormancy and resuscitate, anti-TNF-α antibody treatment, should inhibit Δ*lipY* to resuscitate. To test for this possibility PBMCs from human donors were infected with *Mtb* WT, Δ*lipY* mutant, *or ΔlipY* complemented strain *(ΔlipY C+)* in the *in vitro* model. WT, Δ*lipY* mutant and the *ΔlipY C+* formed micro-granulomas. The granulomas were then treated with either the control IgG or anti-TNFα mAbs for 6 days, and *Mtb* cells were obtained from the granulomas and assessed for dormancy.

**Figure 8:**
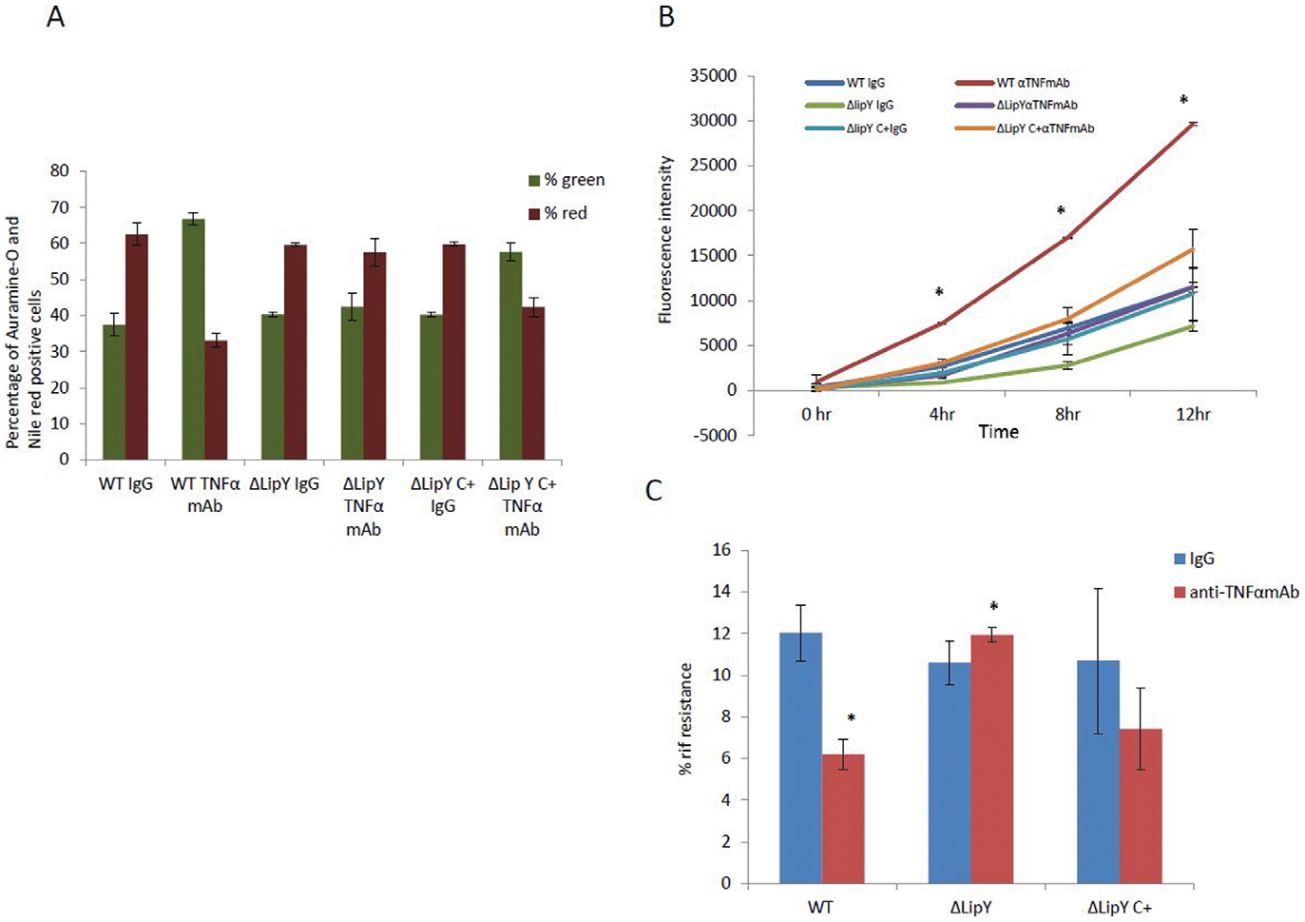
Deletion of *lipY* compromised the ability of *Mtb* to reactivate upon treatment with anti-TNFα mAb. (**A**) Auramine-O and Nile red staining of WT and *Δ-lip Y* mutant and *Δ-lip Y complemented strain (Δ-lip Y C+)* population in *in vitro* grown granuloma. (**B**) Alamar Blue readouts at different time intervals when WT and *Δ-lip Y, and Δ-lip Y C+ Mtb cells* from granulomas treated with anti-TNF-α antibody or control IgG, were incubated with Alamar blue dye. (data of one representative data set has been displayed.) (**C**) *Δ-lip Y* mutant from granulomas treated with IgG or anti-TNF-α antibody had a higher antibiotic resistance as compared to WT *Mtb* cells from anti-TNF-α antibody treated granulomas. Data are presented as mean +/− SEM from 3 experiments. P values were calculated using students t test. *p<0.05.

Auramine-O and Nile red staining of WT *Mtb* cells from granulomas treated with the control IgG, showed that majority of the cells were positive for Nile red and that most of the WT *Mtb* cells from granulomas treated with anti-TNFα mAb were positive for the Auramine-O stain (Fig 8A). Staining of Δ*lipY* mutant *Mtb* cells from granulomas treated with IgG, as well as those from granulomas treated with anti-TNFα mAb showed that most of the cells remained positive for Nile red (Fig. 8A), suggesting that the Δ*lipY* mutant *Mtb* cells could not break dormancy and reactivate, unlike the WT *Mtb*. Staining of Δ*lipY C+Mtb* cells from granulomas treated with anti-TNFα mAb, showed that majority of the cells were positive for the Auramine-O stain, and that most of the Δ*lipY C+Mtb* cells from granulomas treated with control IgG, were positive for Nile red, as observed with the WT *Mtb* cells.

Alamar blue assays suggest that WT *Mtb* cells obtained from granulomas treated with control IgG and Δ*lipY* mutant *Mtb* cells from granulomas treated with IgG or anti-TNFα mAb were less metabolically active as compared to WT *Mtb* from granulomas treated with anti-TNFα mAb (Fig 8B), suggesting that deletion of *lipY* compromised the ability of the pathogen to come out of dormancy. Δ*lipY C+Mtb* cells from granulomas treated with anti-TNFα mAb were more active as compared to the Δ*lipY C+Mtb* cells from granulomas treated with control IgG, although the complementation of Δ*lipY* gene resulted in less increase in metabolic activity as compared to WT after treatment of granulomas with anti-TNFα mAb.

WT *Mtb* from granulomas treated with IgG and Δ*lipY* mutant *Mtb* cells from granulomas treated with IgG or anti-TNFα mAb had a higher percentage of Rif resistance as compared to the WT *Mtb* from granulomas treated with anti-TNFα mAb (Fig 8C), suggesting that Δ*lipY* mutant had a compromised ability to come out of dormancy. Δ*lipY C+Mtb* cells from granulomas treated with anti-TNFα mAb had a lower percentage of Rif resistance as compared to the Δ*lipY C+Mtb* cells from granulomas treated with control IgG, although the difference in percentage of Rif resistance of the Δ*lipY C+Mtb* cells from granulomas treated with control IgG and anti-TNFα mAb, was less as compared to the difference in the percentage of Rif resistance of the WT *Mtb* cells from granulomas treated with control IgG and anti-TNFα mAb. This suggests that the complementation of the *lipY* gene helped the mutant to regain its ability to resuscitate upon treatment with anti-TNFα mAb. However the resuscitation in the complemented strain was less when compared to the WT *Mtb* cells.

## Discussion

We report here, the development of an *in vitro* model of human tuberculosis granuloma that mimics *in vivo* human granuloma. Several *in vitro* granuloma models have been developed in the past, mainly using mycobacteria or mycobacterial antigen-coated beads [8], [39] but they do not truly recapitulate the dormant nature of the mycobacterium within the human TB granuloma.

Dormancy of *Mtb* has been shown to be characterized by a non-replicating state, development of resistance to antibiotic rifampicin [30], loss of acid-fastness and accumulation of lipid bodies [23], [25], [40]. Most *in vitro* models of *Mtb* dormancy are able to induce non-replicating state in mycobacteria but they have not been able to demonstrate all the above characteristics of dormant mycobacteria. Recently, Deb *et. al.* developed a multiple stress model of *Mtb* dormancy in an attempt to replicate the conditions the pathogen is thought to encounter in host granuloma, and showed that *Mtb* indeed exhibited dormancy as characterized by all the hallmark characteristics of dormancy [36], however this model does not involve host-pathogen interaction. In lipid loaded THP-1 derived macrophages *Mtb* has been found to go into a dormant state [13]. At low dose infection, which closely mimics the *in vivo* scenario, *Mtb* in our granuloma model displays characteristics of dormant *Mtb*, *via*. development of Rif-tolerance, loss of acid fastness and accumulation of lipids bodies (Fig 4). These results demonstrate development of *Mtb* latency in our human granuloma model.

The present *in vitro* model and *in vivo* observations in human TB share the following features: granuloma formation, multinucleated giant cell formation, decrease in CD4 T cell counts, unchanged CD8 T cell values, increase in CD4^+^CD25^+^ T cells, decrease in activated macrophage cells, increase in cytokine and chemokine secretion by host immune cells in response to *Mtb* infection, Rifampicin-tolerance, loss of acid fastness and accumulation of lipid bodies, and resuscitation upon immunosuppression by treatment with anti-TNFα mAbs. Below we list features of our in vitro model that are consistent with human findings.

### Microstructure

In our model within 5–8 days post-infection, the lymphocytes in human PBMCs show clustering around infected macrophages resembling micro-granuloma aggregates (Fig 1). The micro-granuloma is formed specifically in response to *Mtb* infection since uninfected samples do not exhibit such aggregates. This model can potentially provide insights into host-pathogen interactions at various stages of human TB granuloma formation which has not been possible with animal models as well as other *in vitro* models. Multinucleated giant cell formation due to fusion of macrophage cells is a characteristic of TB granuloma [8], [41] and our *in vitro* model is able to recapitulate this as observed from the histological sections of the granuloma samples (Fig 1D). Importance of multinucleated giant cell in granuloma and their role can be studied in detail using this model.

### Cellular Responses

The pattern of cell surface marker expression on lymphocytes from the in vitro granuloma model reflects data reported on tuberculosis patients reported in literature [42], [43]. The CD4 T cells were significantly lower in the blood, but CD8 T cells were normal in patients with pulmonary TB when compared with values obtained in normal blood donors. [44] It is reported that TB causes CD4 T cell lymphocytopenia and is associated with normal numbers of CD8 T cells. In our *in vitro* granuloma model, we also observed a similar decline in CD4 T cells while the CD8 T cells and also B cells remain unchanged in the granuloma samples (Fig 2). CD4+25+ regulatory T cells are known to be important in host immune response to TB. There’s reportedly a three-fold increase in the frequency of CD4+CD25 high T cells in blood and disease sites in TB patients [45]. Similarly, we observed several-fold increase in numbers of CD4+25+ regulatory T cells in infected samples compared to uninfected culture samples (Fig 2). It has been reported that there is a decrease in alveolar macrophage numbers in bronchoalveolar lavage fluid obtained from affected regions of pulmonary TB patients compared to non-affected regions of the lungs of patients with active or inactive pulmonary TB [46]. In our flow-cytometric profile of macrophage cells, we also observed a large decrease in macrophage cell numbers and in activated macrophages from the granuloma samples compared to uninfected samples. Macrophages are the primary host cells for *Mtb* and consequently, there is extensive cell death among this population of cells.

### Cytokine Responses

Several cytokines are known to play an important role in anti-TB immunity. Increased mRNA expression of IFN-γ TNF-α, IL-6, IL-8 and IL-12 in tuberculosis granuloma [47] and secretion of cytokines IL-1β, TNF-α and IL-6 in bronchoalveolar lavage fluid from involved sites of pulmonary TB have been reported [48]. In this *in vitro* model, we also observed induction of IFN-γ, TNFα, IL-12p40 and IP-10, in infected samples. Thus, our *in vitro* model is able to faithfully recapitulate the *in vivo* scenario in terms of cytokine and chemokine secretion by host immune cells in response to *Mtb* infection.

### Dormancy

*Mtb* is thought to survive in a dormant state for many years in the host. Under opportune conditions, like immunosuppression or co-infection with infectious agents like HIV, *Mtb* resuscitates to develop active TB. There are widely reported cases of reactivation of latent TB as a result of anti-arthritis treatments based on neutralization of TNF-α or its receptor, eg. Adalimumab, Etanercept or Infliximab. [49], [50] TNF-α is important in development and maintenance of TB granuloma, and is essential for maintaining the state of dormancy [32], [51], [52] [50], [53]–[56]. Neutralizing TNF-α or its activity allows *Mtb* to emerge from dormancy and develop active TB [14]–[16], [18], [32], [33]. Although we did not find any significant difference in the structural morphology of the granulomas treated with a control IgG and anti-TNFα monoclonal antibody, we did find differences in the state of dormancy of the *Mtb* from these granulomas. This is in agreement with non-human primate studies in which it was demonstrated that TNF neutralization reactivates latent infection, but retained normal granuloma structure, despite TNF neutralization during both primary and latent infection [57]. Recently, zebrafish and nonhuman primate models reported that although TNF is important for overcoming acute infection and preventing reactivation, the overall granuloma formation is normal in the absence of TNFα [53], [57], [58].

### Reactivation

It is known that the dormant *Mtb* within the granulomas is in a metabolically inactive state. Under certain conditions, such as treatment with anti-arthritis drugs e.g. Adalimumab, Etanercept or Infliximab, that are based on neutralization of TNF-α or its receptor, the dormant *Mtb* becomes metabolically active, resulting in active TB. In our *in vitro* model, resuscitation of the dormant *Mtb* following treatment of granulomas with anti-TNFα mAb was demonstrated by the Alamar Blue assay data, which showed an increase in metabolic activity of the *Mtb* from anti-TNFα mAb treated granulomas. Furthermore, increased utilization of oleic and palmitic acid by the *Mtb* from anti-TNFα mAb treated granulomas as compared to that of the *Mtb* from the control IgG treated granulomas also demonstrate that the *Mtb* is metabolically active following anti-TNFα monoclonal antibody treatment. Rif tolerance and Auramine-O and Nile red staining data also suggest resuscitation of the *Mtb* following anti-TNFα monoclonal antibody treatment. No other *in vitro* model has demonstrated resuscitation of *Mtb* under immunosuppressive conditions.

### Apoptosis

It has been reported that *Mtb* infection causes apoptosis of monocytes [59], [60]. Our TUNEL assay results also show that infection of PBMC cells with *Mtb* resulted in apoptosis of these cells in our *in vitro* granuloma model. However anti-TNF mAb treatment did not affect apoptosis. Our data correlates to the study that showed that neutralization of secreted TNF-α did not inhibit *Mtb*-induced apoptosis in PBMC from TB-patients [61]. Since *Mtb -*induced apoptosis in is not mediated by the production of TNF-α [61], in our *in vitro* granuloma model, we did not observe any change in the number of apoptotic cells following treatment of granulomas with anti-TNF mAb.

### Gene Expression Profile

To determine whether the dormancy and resuscitation phenotypes observed with the *Mtb* cells from *in vitro* granuloma are reflected in the change in gene expression levels, we measured transcript levels of certain selected genes associated with TG accumulation and catabolism, dormancy and resuscitation. The *Mtb* genes, *tgs1*, *gltA1, citA*, *icl*, *hspX* and *dosR*, that are known to be up regulated under dormancy inducing conditions [13], [35], [36] were more highly induced in IgG treated *Mtb* cells, compared to anti-TNFα-mAb treated *Mtb* cells from the *in vitro* granuloma. This result is consistent with the observed dormancy phenotypes in *Mtb* cells from *in vitro* granuloma. Down-regulation of these genes and up regulation of *lipY* in anti-TNFα-mAb treated *Mtb* cells from granuloma indicate that the *Mtb* cells were under resuscitation state. We previously have shown that *lipY* transcript level goes up when the *Mtb* cells are starved after lipid accumulation under dormancy inducing stress conditions [62]. The up regulation of genes that are involved in resuscitation (*rpfA*, *rpfB* and *rpfC*), energy generation (*nuoA*, *nuoB*, *nuoE*, *atpA*, *atpB*) and transcription (*rpoA*, *rpoB*) strongly suggest that *Mtb* cells inside the granuloma that were dormant prior to the treatment with anti-TNFα-mAb were resuscitated by anti-TNFα-mAb treatment. Thus, this *in vitro* granuloma model using human PBMC is suitable for studying dormancy and resuscitation, mimicking the events that are thought to happen when a person with latent *Mtb* infection is subjected to immunocompromising conditions.

### *Mtb* Mutants

The conclusion that in our *in vitro* granuloma model mimics what happens in vivo is supported by studies on *Mtb* mutants. *tgs1* is known to be involved in lipid accumulation that occur in *Mtb* under different stresses that lead to dormancy [13], [63]. Earlier studies from our lab have shown that the *tgs 1* deletion mutant, with impaired ability to accumulate TG, exhibited a lesser degree of antibiotic tolerance. [36]. In our *in vitro* granuloma model *tgs1* deletion mutant showed compromised ability to go into dormancy There is evidence that *lipY,* the only known mycobacterial enzyme with long chain TG hydrolase activity, is involved in the utilization of stored TG in *Mtb* [62], [64]. It has been suggested that *lipY* is involved in mobilizing stored TG during reactivation. In our human granuloma model the *lipY* deficient *Mtb* demonstrated compromised ability to resuscitate upon immunosuppression with anti-TNFα antibody.

Thus, we believe that the battery of phenotypic and functional assays demonstrate that we have been able to develop an *in vitro* granuloma model of dormancy and resuscitation that accurately reflects human responses. Our granuloma model is able to mimic human micro-granuloma in terms of cellular organization, expression of cellular markers on host cells and development of mycobacterial dormancy. The compromised ability of the *tgs 1* mutant to go into dormancy and the inability of *lipY* mutant to get out of dormancy add further validation to the conclusion that the present model mimics the *in vivo* situation. The most important application of this *in vitro* model may be to provide a platform for testing vaccine and drug candidates against active as well as dormant *Mtb* potentially helping development of new and effective drugs and vaccines against TB. This model can also be used to test for anti-mycobacterial drugs that can kill dormant *Mtb*.

## Materials and Methods

### PBMC Cell Isolation from Human Blood

Human Blood was collected at a blood donation center of the Florida Blood Center in Orlando, FL, from healthy volunteers as per written informed consent. Florida Blood Centers operates under license from the Food and Drug Administration of the US Department of Health and Human Services. Blood collection and processing was done as per approval from IRB which determined that our use of blood from the Blood Center is exempt from our institutional review board. All donors were tested negative for standard panel of blood-borne pathogens tested by the blood center. PBMCs were purified by Ficoll density gradient separation using standard protocol used for separating PBMCs. After washing, PBMCs aliquots of 2×10^7^ cells/vial were cryopreserved in 10% DMSO-containing media for extended storage in liquid nitrogen. When needed, PBMC vials were thawed and then washed in RPMI containing 5% human Serum. Cells were suspended in RPMI containing 20% human serum and counted by trypan blue dye exclusion method.

### *Mtb* Culture

*M. tuberculosis* H37Rv (WT) was cultured in Middlebrook 7H9 medium (supplemented with 10% OADC, 0.2% glycerol and 0.05% Tween 80) (Difco, USA) *Δtgs*1 [63] and Δ*lipY* [*62*] mutants were cultured in the same medium with Hygromycin 75 µg/ml. Δ*tgs1 C+*and Δ*lipY* C+, were cultured in the same medium with Hygromycin 75 µg/ml and kanamycin 20 µg/ml. Cultures containing 2.8×10^8^ cfu/ml *Mtb* was used for all experiments. *Mtb* cells were suspended in 1 ml of RPMI containing 20% human Serum, water bath-sonicated for 2 pulses of 30 s each and used for infection.

### PBMC Infection and Granuloma Formation

An extracellular matrix (ECM) was prepared by mixing 0.95 ml Purecol collagen solution, 50 µl 10×DPBS (Lonza, USA), 4 µl fibronectin (BD Biosciences, USA) and 10 µl 1N NaOH (Sigma, USA) per ml of matrix solution and kept on ice (pH 7.0 ). PBMC cells were mixed at room temperature (RT) with ECM at 5×10^5^cells/50 µl/well of 96-well plate. Assuming 5% macrophages in PBMCs, required strain of *Mtb* was added to the ECM for infected samples at MOI of 0.1. ECM was allowed to set by incubating on 37°C, CO_2_ incubator for 45min. Samples were added with RPMI containing 20% human Serum and incubated in 37°C, CO_2_ incubator. Media was changed on day7.

### Paraffin Embedding, Sectioning and Histological Staining

On day 8 or 9 of incubation, media was removed from granuloma samples and fixed with 4% paraformaldehyde overnight. Samples carefully removed intact from the wells were paraffin-embedded and sectioned, [65] Paraffin sections were deparaffinized and stained by standard acid-fast bacilli (AFB) staining or by Auramine O-Nile Red fluorescent staining. For AFB staining, slides were stained using TB quick stain kit (Becton Dickinson Biosciences, USA) using manufacturer’s protocol.

### Immunohistochemistry for Cell Types in the *in vitro* Granuloma

For immunohistochemistry 0.5 micron meter sections were subjected to immunohistochemistry [65]. Deparaffinized sections were boiled in 10 mM Citrate buffer for 30 min, cooled, and blocked with 5% normal donkey serum in PBST for 40 min. incubated with CD3(Tcell marker) polyclonal rabbit anti-human antibody from Dako overnight. followed by incubation with fluorescent secondary antibody Dylight 488 conjugated affinipure donkey anti-rabbit IgG (green) for 30 minutes, then incubated with CD68 Mouse Monoclonal antibody (macrophage marker) for 60 min. and finally incubated with fluorescent secondary antibody CY3 conjugated affinipure donkey anti-mouse IgG (Red) for 30 min. After washing and dehydrating the slides were mounted with media containing DAPI, and viewed under a Nikon TE2000 florescent microscope (Nikon Corp., Tokyo, Japan). Images were acquired using a coolsnap HQ^2^ camera (Photometrics, Tuscon,AZ) or a Nikon Digital Sight DS Ri1 camera. “NIS elements” software (Nikon) was used for acquisition of images. Images were taken using the Texas red filter set, FITC filter set and the DAPI filter set (Chroma, Rockingham,VT).

### AuramineO-Nile Red Staining

Staining was done using TB Fluorescent Stain Kit M (Becton Dickinson Biosciences, USA), as described [36], [40]. Slides were mounted in cytoseal with coverslip and viewed under the Nikon TE2000 florescent microscope (Nikon Corp., Tokyo, Japan). Images were acquired using a cool snap HQ^2^ camera (Photometric, Tuscon, AZ) or a Nikon Digital Sight DS Ri1 camera. “NIS elements” software (Nikon) was used for acquisition of images. Images were taken using the Texas red filter set, and the FITC filter set (Chroma, Rockingham, VT).

### Rifampicin Treatment and Plating

Rif was dissolved in DMSO, and granuloma samples were either treated with Rif or left untreated (control) (Sigma, USA) (Rif, 5 µg/ml final concentration). Earlier works from our lab have used this concentration of Rif. On day 8- or 9-post-infection, media was removed, replaced with 100 ul/well of media containing Rif and incubated for 3 days. Then, media was removed and wells treated with 50 µl/well collagenase (Sigma, USA) for 40 min at 37°C to isolate host PBMC cells. Samples from five wells were pooled in 1.8 ml micro centrifuge tubes and host cells were lyzed with 200 µl of 0.1% triton X-100 solution. *Mtb* pellet was obtained by centrifuging at 3500×g for 12 min. the *Mtb* pellet was suspended in 1 ml 7H9 media and 10-fold serial dilutions were made in Middlebrooks 7H9 media containing 0.05% tween-80 and 100 µl samples plated on Middlebrooks 7H10 agar plates. Plates were incubated at 37°C. Colony forming units (cfu) were determined after four weeks. % Rifampicin-tolerance is calculated by formula - %Rif-tolerance = cfu(Rif)/cfu(untreated)×100.

### Apoptosis Assay

Nuclear DNA fragmentation consistent with apoptosis was determined by the TUNEL method [66], [67]. We used the CF^TM^594 TUNEL Assay Apoptosis detection kit. (Biotium). TdT catalyzed the addition of dUTP conjugated to red fluorescent dye, onto the 38-OH termini in the DNA of apoptotic cells. DAPI blue counterstain was also used. Media was removed from the wells containing uninfected PBMCs and IgG or anti-TNFα mAB treated granulomas, and they were treated with 50 µl/well collagenase (Sigma, USA) for 40 min at 37°C to isolate host PBMC cells. Samples from five wells were pooled in 1.8 ml micro centrifuge tubes and cells were fixed with 4% paraformaldehyde overnight. A smear of fixed cells was put onto the slide, and were then microwaved for 2 min in 10 mM Citrate buffer. The slides were washed with PBS twice, and then permeabilized in PBS containing 0.2% Triton X-100 for 30 minutes. TUNEL assay was done following the kit protocol. The slides were then mounted with mounting media containing DAPI. Slides were then observed using a Nikon TE2000 florescent microscope (Nikon Corp., Tokyo, Japan). Images were acquired using a cool snap HQ^2^ camera (Photometric, Tuscon, AZ) or a Nikon Digital Sight DS Ri1 camera. “NIS elements” software (Nikon) was used for acquisition of images. Nuclei containing dark red precipitate indicated TUNEL-positive cells. Five to six random fields totaling 500 cells were counted per slide. The percentage of cells undergoing apoptosis is calculated by dividing the number of positive nuclei by the total number of nuclei counted multiplied by 100.

### Bioplex Cytokine Assay

Cytokine/chemokine analysis from granuloma supernatants was done using 22-plex Beadlyte human cytokine/chemokine kit (Millipore, USA) as per manufacturer’s instructions.

### Flow Cytometric Analysis

Host cells were isolated by collagenase treatment of granuloma and uninfected samples as described above. Cells from five similar wells were pooled and washed with 1x PBS. Surface and intracellular staining was done in a seven-color analysis with combinations of fluorescein isothiocyanate (FITC)-, phycoerythrin (PE)-, allophycocyanin (APC)-, peridinum chlorophyll Cy5.5 (PerCP-Cy5.5)-, phycoerythrin Cy7 (PE-Cy7)-, allophycocyanin Cy7 (APC-Cy7)-conjugated antibodies and Annexin V-Pacific Blue. Antibodies used included: anti-CD3 (clone SK7), anti-CD4 (SK3), anti-CD8 (SK1), anti-CD11c (B-ly6), anti-CD19 (HIB19), anti- CD25 (2A3), (Becton Dickinson Biosciences, USA). Flow cytometric data was analyzed using FloJo software (Becton Dickinson, USA).

### Treatment of Granulomas with IgG or anti-TNFα Monoclonal Antibody

After the formation of microgranuloma, anti-TNFα monoclonal antibody or control IgG was added to the media at a concentration of 10 ng/ml.

### Metabolic Activity of *Mtb* Using Alamar Dye

After 6 days of treatment of granulomas with anti-TNFα antibody or control IgG, the *Mtb* cells recovered as indicated above were suspended in media and 100 µl were added to each well of a black bottom microtitre plate. 10 µl of 1∶1000 diluted Alamar blue was added to each well and the increase in fluorescence at 590 nm, after excitation at 530 nm, was monitored at different time intervals after addition of the dye using a BioScan Chameleon V plate reader. Wells containing 100 µl of media and 10 µl of 1∶1000 diluted Alamar blue were used as a control. The Alamar readouts were normalized with cfu counts for each strain and analyzed.

### Metabolism of Oleic and Palmitic Acid

The *Mtb* was obtained from PBMCs as described earlier. In a test-tube the *Mtb* cells were suspended in1ml 7H9 media without glycerol, and 5 µci of [1-^14^C] oleic acid or [1-^14^C] palmitic acid was added to the cells. A thin strip of filter paper dipped in 20%KOH was carefully hung at the mouth of the test-tubes and the tubes were then sealed tightly with rubber stoppers and kept on rollers for 4 hr. After 4 hours, the filter paper was carefully removed and submerged in scintillation fluid and the amount of ^14^CO_2_ was determined by liquid scintillation counting using a tri- Carb 2900 liquid scintillation analyzer (Perkins-Elmer, Waltham,M.A). The dpm was normalized to the cfu counts of *Mtb* from IgG or anti-TNFα antibody treated granulomas and the data is presented as pmoles of ^14^CO_2_ released.

### Gene Expression Analyses of *Mtb* from *in vitro* Granuloma – Infection, RNA Isolation, Multiplex Pre-amplification PCR and TaqMan Real-time PCR

Infection of PBMC to induce granuloma formation was done as described above in this section. After the required period of incubation for granuloma formation and resuscitation media was removed from each well. RLT buffer (RNeasy Kit from Qiagen Inc., USA) containing βmercaptoethanol, and RNase inhibitor was added to each well and incubated for less than 10 min at 37°C to lyse the collagen matrix and release the granuloma and the *Mtb* cells. The lysate was collected in a tube and centrifuged at 3500×g for 12 min. The pellet was resuspended in trizol and snap frozen in liquid nitrogen. It was then stored until further use. RNA isolation, cDNA synthesis, multiplex pre-amplification PCR and TaqMan real-time PCR were performed as we have described earlier [13], [36].

### Accession Numbers

tgs1/Rv3130c, POA650; lipY/Rv3097c, P77909.
